# Acculturation as a Determinant of Obesity and Related Lifestyle Behaviors in a Multi-Ethnic Asian Population

**DOI:** 10.3390/nu15163619

**Published:** 2023-08-17

**Authors:** Su Hyun Park, Yu Qi Lee, Falk Müller-Riemenschneider, Borame Sue Lee Dickens, Rob M. van Dam

**Affiliations:** 1 Saw Swee Hock School of Public Health, National University of Singapore and National University Health System, Singapore 117549, Singapore; ephlyq@nus.edu.sg (Y.Q.L.); falk.m-r@nus.edu.sg (F.M.-R.); ephdbsl@nus.edu.sg (B.S.L.D.); rob.van.dam@nus.edu.sg (R.M.v.D.); 2 Digital Health Center, Berlin Institute of Health, Charité-Universitäts Medizin Berlin, 10117 Berlin, Germany; 3 Department of Exercise and Nutrition Sciences, Milken Institute School of Public Health, The George Washington University, Washington, DC 20052, USA

**Keywords:** acculturation, obesity, diet, physical activity, sedentary behavior, Asians, ethnicity

## Abstract

Limited attention has been given to the role of cultural orientation towards different ethnic groups in multi-ethnic settings without a dominant host culture. We evaluated whether acculturation levels, reflecting cultural orientation towards other ethnic groups, were associated with obesity and related lifestyle behaviors in a cosmopolitan Asian population. We conducted the current study based on data from the Singapore Multi-Ethnic Cohort (*N* = 10,622) consisting of ethnic Chinese, Malays, and Indians aged 21 to 75 years. Multivariable linear and logistic regression analyses were used to examine associations between the acculturation level (z-score), obesity, and related lifestyle behaviors, including dietary habits and physical activity. A higher acculturation level was directly associated with a higher prevalence of obesity among Chinese, whereas an inverse association was found for ethnic Indians, and no significant association in Malays. In ethnic Malays, greater acculturation was significantly associated with higher dietary quality and less sedentary time. Furthermore, a high acculturation level was significantly associated with higher sugar-sweetened beverage consumption and more leisure-time PA in all ethnic groups. Our findings suggest that greater cultural orientation towards other ethnic groups was associated with convergence in obesity levels. More research is required to understand how acculturation affects obesity-related lifestyle factors in multi-ethnic settings.

## 1. Introduction

Obesity is a risk factor for non-communicable diseases, including type 2 diabetes (T2D), cardiovascular disease (CVD), and several types of cancer [1]. The determinants of obesity are complex, multifaceted, and disproportionate; certain groups are more at risk than others, depending on socioeconomic status [2], ethnicity [3], and the environment [4]. 

Ethnic differences in obesity have been reported in various populations, especially in Western countries, and the differences are not fully explained by individual socioeconomic status (SES) reflected by income or education, or other environmental factors [5,6,7,8,9]. For example, the higher obesity risk for Blacks compared to Whites in the US persists after controlling for socioeconomic status, and even the disparities are largest among those with higher incomes and educational levels [6]. This suggests that the interrelationships among race, SES, and obesity are complex; additional, unmeasured factors such as sociocultural factors, stress, or discrimination may be in play. Few studies have examined the relationship between ethnicity and obesity among ethnic Asians, and most of these studies have reported disparities between Asian subgroups in Western countries [5,10,11]. 

Acculturation, or the cultural and psychological changes that occur when individuals interact with a host or dominant culture [12] has been suggested to contribute to ethnic differences in obesity prevalence. This may reflect that sociocultural factors that are part of ethnic identities can affect obesity-related behaviors such as physical activity and diet [7,13,14,15,16]. In understanding the process of acculturation, Social Identity Theory, a psychological framework proposing that individuals modify their behaviors based on their group affiliations [17] may be helpful. As individuals engage with members of the dominant culture, their social identity may change, leading them to adapt behaviors to align with the values and norms of the host culture for a sense of belonging and group cohesion. The associations between acculturation and obesity in Western countries such as the US and Australia are relatively consistent. Specifically, immigrants exhibit lower body mass indices than those of host country-born subjects, and longer residence and higher acculturation are associated with the development of obesity [18,19,20,21,22].

However, less is known about the associations between acculturation and health outcomes in multicultural settings with distinct cultures, that is, without a dominant host culture, which may be associated with more complex interethnic relationships and dynamics. For example, Singapore is a multiethnic city-state with three major ethnic groups: Chinese, Malays, and Indians. Previous research identified large ethnic differences in obesity; Malays and Indians exhibit a higher prevalence of obesity than Chinese, and this is not completely explained by measures of individual or neighborhood SES [23]. Further research on the underlying sociocultural and contextual factors affecting obesity-related lifestyle behaviors across ethnic groups is thus warranted. 

Therefore, the purpose of this study is to examine the association between acculturation levels and obesity in three Asian ethnic groups residing in Singapore. We also examined the association between acculturation level and obesity-related behaviors, including dietary habits and physical activity. In this context, acculturation refers to the cultural orientation towards other ethnic groups rather than adaptation to a single dominant culture. We expected that greater acculturation results in a body mass index (BMI) more similar to other ethnic groups. Specifically, we hypothesized that greater acculturation is associated with a higher BMI in ethnic groups with a low average BMI and a lower BMI in groups with a high average BMI. 

## 2. Materials and Methods

### 2.1. Study Population

We used cross-sectional data from the follow-up of the Singapore Multi-Ethnic Cohort Phase 2 (MEC2), a population-based cohort study of Singapore citizens and permanent residents aged 21–75 years with three major ethnic groups: Chinese, Malay, and Indian. Detailed information on the MEC2 and its follow-up can be found at https://blog.nus.edu.sg/sphs/the-first-sphs-follow-up/ (accessed on 27 July 2023) and in a previous publication [24]. Data collection of the MEC2 follow-up consisted of a home interview and a physical examination at the health screening center. Typically, the interviews were conducted at the participant’s home or occasionally at another location of their preference. The present study included a total of 13,052 participants. Of these, we excluded those who are not ethnic Chinese, Malays, or Indians (*n* = 20), and those with a history of stroke (*n* = 32), cancer (*n* = 184), or heart attack (*n* = 82). Those who do not have information on BMI (*n* = 1020), acculturation scale (*n* = 890), or dietary intake (*n* = 205) were excluded. We further excluded participants with BMI < 15 kg/m^2^ or >60 kg/m^2^ as outliers (*n* = 47), yielding our final sample of 10,575 participants for the current analysis. Informed consent was obtained from all participants before study enrolment, and the study protocol was approved by the Institutional Review Board of the National University of Singapore (NUS-IRB-reference B-16-125). 

### 2.2. Data Collection and Measurements

Data were collected through face-to-face interviews using standardized questionnaires on computer tablets by trained interviewers. The questionnaire included questions on sociodemographic characteristics such as age, gender, household income, education level and marital status, personal and family history, medication use, lifestyle factors, immigration generation, and acculturation. Information on ethnicity was recorded by an interviewer based on their identity card. 

Subsequently, participants were invited to attend a health screening. Anthropometric measurements, including height and weight, were obtained from participants using WHO standard procedures by trained personnel at the health screening sites. Participants removed their shoes before measuring their height using a portable stadiometer (SECA 200 series), with the head positioned in the Frankfurt plane. Any heavy belongings were removed from their clothing before measuring their body weight using a digital scale (SECA 700 series). BMI was calculated by dividing weight (kg) by height squared (m^2^). In the present study, we defined obesity as a BMI of 27.5 kg/m^2^ or higher based on the WHO cut-off for Asians, as Asians tend to have more body fat for a given BMI compared to persons of European descent [25]. 

Previously, we adapted and validated the acculturation scale, using the 12-item SAS for Hispanics, originally developed to assess the level of acculturation of Hispanics in the U.S. and widely used for Asians in the US [26]. Based on the confirmatory factor analyses (CFA), our adapted version was found to be valid and reliable in measuring the acculturation level in the multicultural Singapore population [26]. Our scale includes three subscales with 11 items: language use (5 items), medical use (3 items), and ethnic social relations (3 items). We summed up 11 items to get a total score. Due to the multilingual setting of Singapore, where more than two languages are relevant, three additional questions were included for each language and media use item. This accounts for the mother tongue language, other Asian languages, and English. The possible range for the total score was from 27 to 135. Higher scores indicate higher levels of acculturation. The questionnaire and scoring method can be found in Park et al. [26] and Table S1.

Dietary intakes were assessed using a validated, semi-quantitative food frequency questionnaire (FFQ) with 163 items, and by asking additional questions on food subtypes and cooking methods [27]. Participants were asked to consider their food intake over the past year. A visual aid was provided (by trained interviewers) to assist participants in quantifying standard portion sizes [27]. Participants reported consumption of standard servings as times per day, week, or month. Items consumed less than once per month were coded as rarely/never. Dietary food intakes were standardized to daily frequencies and multiplied by standard serving sizes (grams). A nutrient database for the FFQ was constructed using the nationally representative 24-h dietary recall data used for FFQ development. Details of the assessment of dietary intake have been published elsewhere [27]. We evaluated the healthfulness of the overall diet using the Dietary Approaches to Stop Hypertension (DASH) score. The score was calculated based on quintiles of intake in the population, assigning a score from 1 to 5 for each component. A higher DASH score indicates that individuals are in higher quintiles of whole grain, fruit, vegetable, nut and legume, and dairy intake, while being in lower quintiles of sugar-sweetened beverage and red meat intake. The scores for each component were added together, resulting in a DASH score ranging from 7 to 35, which reflects the overall healthiness of the diet [28].

Physical activity was assessed using the validated Singapore Prospective Study Program Physical Activity Questionnaire (SP2PAQ), which includes questions on the duration and frequency of a wide range of leisure time activities [29]. We focused on leisure time activity of moderate-to-vigorous intensity for the current study. The metabolic equivalent task units (METs) and the energy expenditures were calculated for each activity type (by duration) using the compendium of Ainsworth et al. [30]. The time spent sitting during daily leisure time was also calculated by collating the time spent sitting during free time on, weekdays and weekends [31]. 

### 2.3. Statistical Analyses

Descriptive statistics (means with SDs, or proportions) were calculated for all variables. Differences were compared between the three ethnic groups using the Kruskal–Wallis and Pearson chi-squared tests. Acculturation scores were converted to standard z-scores before inclusion in multivariable regression analyses. Multivariable linear regression analyses were performed to examine associations between acculturation and BMI, dietary behavior, sedentary time, and physical activity during leisure time. We consider the total acculturation score and each subscale (language use, media use, and ethnic social relations) for the analyses. Multivariable logistic regression analyses were used to examine the associations between acculturation and obesity. We adjusted for age, gender (male vs. female), marital status (single, married, and divorced/separated/widowed), income (less than $2000, $2000–3999, $4000–5999, $6000 or higher, and refused to answer/don’t know), education level (primary or lower, secondary, post-secondary, and university or above) and generation (first, second, and third or older generation). All analyses were stratified by ethnicity as we hypothesized a priori that the impact of acculturation would differ by ethnic group. The interaction between acculturation score and gender was assessed for each outcome variable by adding multiplicative interaction terms to the multivariable analyses. Based on the interaction test results, we employed gender-stratified, multivariable associations of acculturation with the DASH diet score, sedentary time, and leisure-time physical activity. All statistical analyses were performed using Stata 14 (Stata Corp, College Station, TX, USA), and *p*-values < 0.05 were considered statistically significant.

## 3. Results

### 3.1. Participants’ Characteristics

The characteristics of the 10,575 participants included in our analysis are presented in Table 1. The mean age was 49.6 (SD 13.4) years and 56.6% were female. Ethnic Chinese constituted the largest ethnic group (70.6%), followed by ethnic Indians (17.0%) and ethnic Malays (12.4%). The average BMI was 24.7 (SD 4.7) kg/m^2^, and the prevalence of obesity was 22.0%. Regarding the acculturation level, the total acculturation scores were highest for ethnic Indians, followed by ethnic Chinese and Malays. We found significant differences in obesity and related lifestyle variables between the ethnic groups. The prevalence of obesity was highest in Malay (42.5%), intermediate in Indian (35.0%), and lowest in Chinese (15.3%) participants. The DASH dietary quality score and leisure physical activity were highest for ethnic Indians, whereas the consumption of sugar-sweetened beverages (SSB) and fried food, and sedentary time were highest for ethnic Malays.

### 3.2. Associations between Acculturation and Obesity 

The associations between acculturation and obesity among ethnic groups adjusted for age, gender, marital status, income level, education level, and generation are shown in Table 2. Ethnic Indians who were more acculturated had a lower BMI (β −0.43 kg/m^2^; 95% CI −0.70–−0.17 per SD) and lower odds of obesity (OR 0.83; 95% CI 0.73–0.93 per SD), and this was consistent across different subscales of acculturation except for ethnic social relation scores. In contrast, ethnic Chinese with higher acculturation scores had a higher BMI (β 0.16 kg/m^2^; 95% CI 0.06–0.25 per SD) and greater odds of obesity (OR 1.07; 95% CI 1.00–1.15 per SD). No significant association was found in ethnic Malays.

### 3.3. Associations between Acculturation and Related Lifestyle Behaviors 

Table 3 shows the multivariable-adjusted associations between acculturation level and related dietary and movement behaviors. In all ethnic groups, higher acculturation was associated with higher SSB consumption and more leisure physical activity. In ethnic Malays, higher acculturation was also associated with better DASH scores and less sedentary time. In contrast, among ethnic Chinese, higher acculturation was also associated with higher fried food consumption. The results were generally consistent for different acculturation subscales. However, in Chinese participants, ethnic–social relationships were more strongly associated with a higher DASH score, more physical activity, and less sedentary time than other aspects of acculturation. 

As significant interactions with gender were apparent in terms of the associations of the total acculturation score with BMI (*p* = 0.02 among ethnic Malays), sedentary time (*p* = 0.001 among ethnic Malays), and leisure-time physical activity (*p* < 0.0001 in the total sample), we conducted additional analyses stratified by gender (Table S2). Generally, acculturation level was more strongly associated with lifestyle behaviors in men compared with women. The association between higher acculturation and less sedentary time was stronger in Malay men than in Malay women. Although we did not find a significant association between BMI and acculturation in Malays, a direct association was observed in Malay men. In Chinese men, higher acculturation scores were more strongly associated with higher BMI, DASH diet scores, and leisure-time physical activity than in Chinese women. The exception, however, was that, in Indians, the inverse association between acculturation and BMI were stronger in women.

## 4. Discussion

We assessed the level of acculturation and examined its associations with obesity, and movement and dietary behaviors in a multi-ethnic setting with no dominant culture. In this context, acculturation reflects the level of orientation toward the cultures of other ethnic groups rather than the adoption of a dominant host culture. Acculturation frameworks such as the one proposed by Berry [12] are mostly focused on the extent to which immigrants adopt a host culture. Our findings suggest that application to contemporary multicultural societies may require expansion of these frameworks to incorporate bidirectional cultural exchange between ethnic groups. Furthermore, globalization may lead to additional cultural changes, for example, as a result of exposure to Western media and foods. In line with our hypothesis, greater acculturation was associated with a higher BMI and odds of obesity in ethnic Chinese, and a lower BMI and odds of obesity in ethnic Indians. Ethnic Chinese have a lower and ethnic Indians have a higher BMI than the average Singapore population. Thus, a higher acculturation level was associated with a BMI level of ethnic Chinese and Indian participants that was more similar to the BMI of other ethnic groups. However, in ethnic Malays, acculturation was not associated with BMI levels. There was also evidence of an association between greater acculturation and convergence of lifestyle habits with those of other ethnic groups. Specifically, in ethnic Chinese, higher acculturation was associated with more fried food consumption and, in Malays, higher acculturation was associated with a higher DASH score and less sedentary time. However, in all ethnic groups, greater acculturation was associated with higher SSB consumption and more leisure time physical activity. These associations may reflect a stronger orientation towards Western culture as our instrument does not distinguish between orientation towards other Asian or Western cultures.

The direct association between acculturation and obesity among ethnic Chinese agrees with research conducted in Western countries [32,33,34]. This association may occur because their Eastern/traditional eating patterns (i.e., more vegetables and grains) are healthier than the Western diet of more processed, high-fat, and sugary foods that are readily available or convenient to prepare [35,36]. Moreover, our findings of high SSB and fried food consumption support this association between acculturation and obesity, as also reported in high-income countries such as the United States and Australia [36,37,38,39]. Lee et al. (2022) suggested that, although their diet changed substantially after migration, Chinese people tend to incorporate Western-style foods for convenience and maintain their traditional Chinese diet rather than changing the diet completely, especially in multicultural societies [40]. As dietary acculturation is complex, i.e., is affected by personal, cultural, and environmental factors [41], it may be necessary to consider the overall context of acculturation (i.e., family composition or food environment) to accurately identify how dietary acculturation influences unhealthy or healthy dietary changes [42].

Ethnic Indians had the highest overall acculturation scores among the three ethnic groups in this study. Moreover, unlike ethnic Chinese, greater acculturation was associated with a lower prevalence of obesity in ethnic Indians. Previous results regarding the relationship between the level of acculturation and obesity in ethnic Indians have been mixed [43,44,45]. South Asian immigrants in Canada reported an overall positive change in their dietary practices, such as increased fruit and vegetable consumption and healthier food preparation practices, after immigration due to increased nutritional knowledge and awareness [45]. A US study reported that stronger traditional cultural beliefs were associated with higher odds of consuming a ‘fried snacks, sweets, and high-fat dairy’ pattern and with lower odds of consuming an ‘animal protein’ pattern among South Asians [46]. The discrepancy could be due to multiple factors, including sociocultural interactions between the host country and their country of origin, the food environment, and media exposure [47]. We also found that more accultured Indians engaged in more leisure time PA and consumed more SSB, suggesting that other cultural influences can have both negative and positive effects depending on the specific lifestyle behavior.

In ethnic Malays, who had the highest BMI among the three ethnic groups, we observed no association between acculturation and obesity. However, more beneficial than detrimental associations were found between acculturation levels and lifestyle behaviors, such as less sedentary time, more leisure-time PA, and higher DASH scores. The exception was SSB consumption, which was directly associated with acculturation level. A possible explanation for the lack of association between acculturation and obesity in ethnic Malays may be a role of religion, as more than 90% of Malays in Singapore are Muslims. Religious practices and perceptions can affect lifestyle behaviors, such as halal dietary restrictions, and possible hurdles to PA (e.g., women not being able to exercise in the presence of men), especially in women [48]. Comprehensive assessments of acculturation coupled with qualitative research may help understand the possible interplay between religion and acculturation in the development of obesity.

We found that greater acculturation was associated with high SSB consumption and more physical activity in all three ethnic groups in Singapore. Previous studies also reported a direct association between acculturation and SSB consumption in various populations in Western countries [45,49,50], suggesting that higher SSB consumption is strongly associated with greater orientation to Western culture. Further research should investigate other factors moderating the association between acculturation and SSB consumption, including interpersonal and environmental factors. In terms of PA, more-acculturated individuals engaged in more leisure time PA than less-acculturated individuals. Inconsistent findings have been reported regarding the relationship between acculturation and PA. A longitudinal study of Hispanic and Asian-American adolescents reported that acculturation to the US was associated with less PA [51]. The opposite was reported in another sample of Hispanic/Latino adults; accultured Latinos were more likely to participate in leisure-time PA [52]. A more recent study using both self-report and accelerometer-based measures of PA found that those who had been living in the United States for longer participated in more accelerometer-based moderate-to-vigorous intensity PA, and higher social acculturation was associated with more self-reported leisure time PA [53]. Despite more leisure time PA, more accultured Chinese were at greater risk of obesity, highlighting the multifactorial etiology of obesity, which may be related to dietary factors such as higher consumption of fried food and SSB. 

This study had some limitations. First, we used cross-sectional data, so we cannot establish the direction of causal effects between acculturation and obesity-related behaviors. Furthermore, we evaluated several obesity-related lifestyle behaviors, but other relevant risk factors of obesity should also be studied, such as sleep health, depression, and stress. Although we used validated standardized measurements of the levels of acculturation, dietary intake, and PA, the results remain subject to measurement error due to the use of self-reported measures. As cultural and environmental factors play a significant role in the relationship between acculturation and obesity, caution is needed when generalizing our findings to other multi-ethnic settings. 

## 5. Conclusions

Our findings suggest that the association between acculturation and obesity differs by ethnic group in a multicultural setting with no dominant culture. Furthermore, acculturation level was associated with both healthy (i.e., increased leisure time PA) and unhealthy (i.e., SSB consumption) lifestyle factors. Given that acculturation is a complex multidimensional process involving personal, sociocultural, and environmental factors, we should interpret the results with caution. This line of research can offer practical implications for public health interventions. Understanding the role of cultural determinants of obesogenic behaviors, and obtaining detailed information on adopting aspects of different cultures and maintaining traditional values may aid the design of more culturally sensitive interventions for obesity prevention. Moreover, our research contributes to the enrichment of the existing theoretical framework for understanding acculturation processes in multicultural settings. Multi-ethnic populations with bidirectional cultural influences are increasingly common in urban settings worldwide. Therefore, future research to further elucidate the ways acculturation affects obesity-related behaviors in multicultural settings is warranted.

## Figures and Tables

**Table 1:** Participant characteristics and acculturation level by ethnicity (*N* = 10,622).

	Total, *n* (%)	Ethnicity			
		Chinese (*n* = 7469)	Malay (*n* = 1310)	Indian (*n* = 1796)	*p*-Value ^a^
**Age (years, mean, SD)**	49.6 (13.4)	51.1 (13.5)	45.6 (12.6)	46.4 (12.4)	0.0001
**Gender**					
Male	4593(43.4)	3229 (43.2)	531 (40.5)	833 (46.4)	0.004
Female	5982 (56.6)	4240 (56.8)	779 (59.5)	963 (53.6)	
**Marital status**					
Single	1744 (16.5)	1376 (18.4)	181 (13.8)	187 (10.4)	<0.0001
Married	7771 (73.5)	5340 (71.5)	1001 (76.4)	1430 (79.6)	
Divorced/separated/widowed	1051 (10.0)	745 (10.0)	127 (9.7)	179 (10.0)	
**Education**					
Primary or lower	2346 (22.1)	1758 (23.5)	292 (22.3)	265 (14.8)	<0.0001
Secondary	2529 (23.8)	1733 (23.2)	429 (32.8)	356 (19.8)	
Post-secondary	2835 (26.7)	1861 (24.9)	468 (35.7)	502 (28.0)	
University or above	2905 (27.4)	2111(28.3)	120 (9.2)	673 (37.5)	
**Monthly household income (SGD)**					
Less than $2000	1843 (17.4)	1334 (17.9)	275 (21.0)	234 (13.0)	<0.0001
$2000–$3999	2104 (19.9)	1315 (17.6)	389 (29.7)	400 (22.3)	
$4000–$5999	2035 (19.2)	1318 (17.7)	264 (20.2)	453 (25.2)	
$6000 or higher	3297 (31.2)	2490 (33.3)	248 (18.9)	559 (31.1)	
Refused to answer/don’t know	1296 (12.3)	1012 (13.6)	134 (10.2)	150 (8.4)	
**Generation**					
First generation	3389 (32.1)	2411 (32.3)	104 (7.9)	874 (48.7)	<0.0001
Second generation	3342 (31.6)	2443 (32.7)	377 (28.8)	522 (29.1)	
Third or older generation	3844 (36.4)	2615 (35.0)	829 (63.3)	400 (22.3)	
**Acculturation score,** **median (IQR)**					
Total score	70.0 (59.0, 82.0)	69.0 (57.0, 82.0)	69.0 (61.0, 77.0)	74.0 (63.0, 85.0)	0.0001
Language use	38.0 (32.0, 45.0)	38.0 (31.0, 46.0)	35.0 (31.0, 41.0)	40.0 (33.5, 47.0)	0.0001
Media use	25.0 (21.0, 30.0)	25.0 (21.0, 30.0)	26.0 (22.0, 29.0)	26.0 (21.0, 29.0)	0.10
Ethnic social relations	6.0 (4.0, 8.0)	5.0 (3.0, 7.0)	8.0 (6.0, 9.0)	8.0 (6.0, 9.0)	0.0001
**Dietary intake, mean (SD)**					
The DASH dietary quality score	21.1 (4.5)	21.0 (4.5)	19.8 (4.4)	22.6 (4.4)	0.0001
Sugary beverages (g/day)	175.0 (255.3)	150.0 (214.6)	334.5 (396.4)	162.6 (237.9)	0.0001
Fried foods (g/day)	34.9 (40.0)	29.9 (34.7)	55.1 (53.3)	40.8 (43.8)	0.0001
**Other lifestyle behaviors, mean (SD)**					
Leisure moderate-to-vigorous physical activity (MET-hr/wk)	16.4 (22.7)	16.0 (21.1)	16.5 (28.1)	18.1 (24.6)	0.0001
Sitting during leisure time (hr/day)	3.2 (2.1)	3.2 (2.1)	3.4 (2.2)	3.0 (1.9)	0.0001
**BMI, kg/m^2^ (mean, SD)**	24.7 (4.7)	23.8 (4.0)	27.4 (5.7)	26.6 (4.9)	0.0001
**Obesity**					
BMI < 27.5 kg/m^2^	8246 (78.0)	6326 (84.7)	753 (57.5)	1167 (65.0)	<0.0001
BMI ≥ 27.5 kg/m^2^	2329 (22.0)	1143 (15.3)	557 (42.5)	629 (35.0)	

^a^ Kruskal–Wallis test or chi-square test.

**Table 2:** Multivariable-adjusted association between acculturation level ^a^ and body mass index (BMI) and odds ratios (ORs) of obesity according to ethnicity.

	Chinese (*n* = 7469)	Malays (*n* = 1310)	Indians (*n* = 1796)
	Beta (95% CI)	Beta (95% CI)	Beta (95% CI)
**BMI (kg/m^2^)**			
Overall acculturation score	**0.16 (0.06, 0.25)**	0.07 (−0.37, 0.51)	**−0.43 (−0.70, −0.17)**
Language use	**0.17 (0.08, 0.27)**	0.11 (−0.33, 0.55)	**−0.44 (−0.70, −0.18)**
Media use	0.08 (−0.01, 0.17)	0.11 (−0.33, 0.54)	**−0.29 (−0.57, −0.02)**
Ethnic social relations	**0.16 (0.05, 0.27)**	−0.14 (−0.46, 0.18)	**−0.23 (−0.45, −0.01)**
**Obesity**	OR (95% CI)	OR (95% CI)	OR (95% CI)
Overall acculturation score	**1.07 (1.00, 1.15)**	1.02 (0.87, 1.19)	**0.83 (0.73, 0.93)**
Language use	**1.08 (1.01, 1.15)**	1.01 (0.87, 1.19)	**0.83 (0.74, 0.93)**
Media use	1.03 (0.97, 1.10)	1.05 (0.90, 1.23)	**0.86 (0.76, 0.97)**
Ethnic social relations	1.08 (1.00, 1.16)	0.96 (0.85, 1.08)	0.94 (0.86, 1.04)

CI, confidence interval; OR, odds ratio. Acculturation raw scores were converted into standard z-scores and beta coefficients were calculated for a 1-SD increase in acculturation scores. ^a^ Adjusted for age, gender, marital status, income level, education level, and generation in Singapore.

**Table 3:** Multivariable-adjusted associations between acculturation level and dietary and movement behaviors according to ethnicity.

	Chinese (*n* = 7469)	Malays (*n* = 1310)	Indians (*n* = 1796)
	Beta (95% CI) ^a^	Beta (95% CI) ^a^	Beta (95% CI) ^a^
**The DASH diet score**			
Overall score	0.06 (−0.05, 0.16)	**0.63 (0.31, 0.96)**	0.19 (−0.03, 0.42)
Language use	0.07 (−0.03, 0.17)	**0.46 (0.14, 0.78)**	0.16 (−0.06, 0.39)
Media use	−0.04 (−0.14, 0.06)	**0.67 (0.35, 0.99)**	0.19 (−0.05, 0.43)
Ethnic social relations	**0.28 (0.16, 0.40)**	**0.29 (0.06, 0.53)**	0.10 (−0.08, 0.29)
**Sugary beverages (g/day)**			
Overall score	**12.38 (7.42, 17.35)**	**39.10 (9.45, 68.75)**	**12.60 (0.37, 24.84)**
Language use	**11.13 (6.18, 16.08)**	27.26 (−4.41, 54.92)	11.81 (−0.22, 23.84)
Media use	**8.38 (3.61, 13.14)**	**39.64 (10.04, 69.24)**	9.17 (−3.62, 21.97)
Ethnic social relations	**16.90 (11.21, 22.60)**	**27.04 (5.33, 48.75)**	8.99 (−1.06, 19.03)
**Fried food (g/day)**			
Overall score	**1.31 (0.52, 2.10)**	0.56 (−3.50, 4.63)	0.27 (−2.07, 2.61)
Language use	**1.09 (0.30, 1.87)**	0.63 (−3.43, 4.69)	0.18 (−2.13, 2.48)
Media use	**0.99 (0.24, 1.75)**	−0.17 (−4.22, 3.89)	0.98 (−1.47, 3.43)
Ethnic social relations	**1.83 (0.93, 2.74)**	0.88 (−2.09, 3.85)	−0.77 (−2.70, 1.15)
**Sedentary time (hr/day)**			
Overall score	0.03 (−0.02, 0.08)	**−0.39 (−0.56, −0.22)**	0.02 (−0.08, 0.13)
Language use	0.03 (−0.02, 0.07)	**−0.49 (−0.66, −0.33)**	0.05 (−0.05, 0.15)
Media use	**0.06 (0.01, 0.11)**	−0.11 (−0.28, 0.06)	0.03 (−0.08, 0.14)
Ethnic social relations	**−0.13 (−0.18, −0.07)**	**−0.17 (−0.29, −0.05)**	−0.07 (−0.16, 0.01)
**Leisure-time physical activity (MET-hr/wk)**			
Overall score	**0.65 (0.14, 1.16)**	**6.30 (4.23, 8.38)**	**3.15 (1.84, 4.45)**
Language use	0.44 (−0.07, 0.94)	**5.97 (3.90, 8.05)**	**2.91 (1.62, 4.19)**
Media use	0.33 (−0.15, 0.82)	**3.61 (1.51, 5.70)**	**2.23 (0.86, 3.60)**
Ethnic social relations	**2.04 (1.46, 2.62)**	**4.36 (2.84, 5.89)**	**2.36 (1.28, 3.43)**

CI, confidence interval; OR, odds ratio. ^a^ Adjusted for age, gender, marital status, income level, education level, and generation in Singapore. Acculturation scores were converted into standard z-scores and beta coefficients were calculated for a 1-SD increase in acculturation scores.

## Data Availability

For data access, researchers can contact the Saw Swee Hock School of Public Health, National University of Singapore (https://blog.nus.edu.sg/sphs/data-and-samples-request/ accessed on 10 September 2020).

## Supplementary Materials

The following supporting information can be downloaded at: https://www.mdpi.com/article/10.3390/nu15163619/s1, Table S1. The adapted Singaporean version of Short Acculturation Scale (SAS) for Hispanic, Table S2. Multivariable associations ^a^ between acculturation level and dietary behaviors stratified by gender.
