# Supplementary material for: Acculturation as a Determinant of Obesity and Related Lifestyle Behaviors in a Multi-Ethnic Asian Population
Source: Nutrients. 2023 Aug 17;15(16):3619. doi: 10.3390/nu15163619 (PMC10459113; doi:10.3390/nu15163619)
Supplement: Supplementary file 1 [file nutrients-15-03619-s001.zip › nutrients-2553386-supplementary.pdf]

Table S1. The adapted Singaporean version of Short Acculturation Scale (SAS) for Hispanic

Subscales	Items	Response
Language use	1. In what language(s) do you think?	
	a) How often do you think in your mother tongue (non-English)? (reverse-coded)	1. Always; 2. Often; 3. Sometimes; 4. Rarely; 5. Never (reverse-coded)
	b) How often do you think in your Other Asian languages?	1. Never; 2. Rarely; 3. Sometimes; 4. Often; 5. Always
	c) How often do you think in English?	
	2. In general, what language(s) do you read and speak?	
	a) How often do you read and speak in your mother tongue (non-English)? (reverse-coded)	1. Always; 2. Often; 3. Sometimes; 4. Rarely; 5. Never (reverse-coded)
	b) How often do you read and speak in your Other Asian languages?	1. Never; 2. Rarely; 3. Sometimes; 4. Often; 5. Always
	c) How often do you read and speak in English?	
	3. What was the language(s) you used as a child?	
	a) When you were a child, how often you used in your mother tongue (non-English)? (reverse-coded)	1. Always; 2. Often; 3. Sometimes; 4. Rarely; 5. Never (reverse-coded)
	b) When you were a child, how often you used in your Other Asian languages?	1. Never; 2. Rarely; 3. Sometimes; 4. Often; 5. Always
	c) When you were a child, how often you used in English?	
	4. What language(s) do you usually speak at home?	
	a) How often do you speak at home in your mother tongue (non-English)? (reverse-coded)	1. Always; 2. Often; 3. Sometimes; 4. Rarely; 5. Never (reverse-coded)
	b) How often do you speak at home in your Other Asian languages?	1. Never; 2. Rarely; 3. Sometimes; 4. Often; 5. Always
	c) How often do you speak at home in English?	
	5. What language(s) do you usually speak with your friends?	
	a) When you speak with your friends, how often you used your mother tongue (non-English)? (reverse-coded)	1. Always; 2. Often; 3. Sometimes; 4. Rarely; 5. Never (reverse-coded)
	b) When you speak with your friends, how often you used your Other Asian languages?	1. Never; 2. Rarely; 3. Sometimes; 4. Often; 5. Always
	c) When you speak with your friends, how often you used English?	
Media use	6. In what language(s) are the TV programs you usually watch?	
	a) How often do you watch TV programs in your mother tongue (non-English)? (reverse-coded)	1. Always; 2. Often; 3. Sometimes; 4. Rarely; 5. Never (reverse-coded)
	b) How often do you watch TV programs in your Other Asian languages?	

	c) How often do you watch TV programs in English?	1. Never; 2. Rarely; 3. Sometimes; 4. Often; 5. Always
	7. In what language(s) are the newspapers or magazines you usually read?	
	a) How often do you read newspapers or magazines in your mother tongue (non-English)? (reverse-coded)	1. Always; 2. Often; 3. Sometimes; 4. Rarely; 5. Never (reverse-coded)
	b) How often do you read newspapers or magazines in your Other Asian languages?	1. Never; 2. Rarely; 3. Sometimes; 4. Often; 5. Always
	c) How often do you read newspapers or magazines in English?	
	8. In general, in what language(s) are the movies you prefer to watch and listen to?	
	a) How often do you watch movies in your mother tongue (non-English)? (reverse-coded)	1. Always; 2. Often; 3. Sometimes; 4. Rarely; 5. Never (reverse-coded)
	b) How often do you watch movies in your Other Asian languages?	1. Never; 2. Rarely; 3. Sometimes; 4. Often; 5. Always
	c) How often do you watch movies in English?	
Ethnic social relations	9. Your close friends are?	1. All from your ethnicity; 2. More from your ethnicity; 3. About half from your ethnicity and half from other ethnicities; 4. More from other ethnicities; 5. All from other ethnicities
	10. You prefer going to social gatherings/parties at which people are?	
	11. The persons you visit or who visit you are?	

Table S2. Multivariable associations[a] between acculturation level and dietary behaviors stratified by sex

	Men			Women		
	Chinese (n=3,229)	Malays (n=531)	Indians (n=833)	Chinese (n=4,240)	Malays (n=779)	Indians (n=963)
	Beta (95% CI)	Beta (95% CI)	Beta (95% CI)	Beta (95% CI)	Beta (95% CI)	Beta (95% CI)
**BMI**						
Overall score	**0.21 (0.06, 0.36)**	0.39 (-0.24, 1.01)	-0.34 (-0.72, 0.05)	0.12 (-0.002, 0.25)	0.01 (-0.60, 0.61)	**-0.50 (-0.87, -0.13)**
Language use	**0.22 (0.07, 0.36)**	0.08 (-0.51, 0.68)	-0.24 (-0.61, 0.13)	**0.14 (0.02, 0.27)**	0.24 (-0.38, 0.86)	**-0.58 (-0.94. -0.21)**
Media use	**0.14 (0.003, 0.29)**	**0.74 (0.10, 1.39)**	-0.39 (-0.79, 0.02)	0.05 (-0.08, 0.17)	-0.10 (-0.69, 0.49)	-0.23 (-0.61, 0.15)
Ethnic social relations	0.13 (-0.03, 0.30)	0.18 (-0.27, 0.64)	-0.19 (-0.49,0.12)	**0.15 (0.002, 0.30)**	-0.29 (-0.72, 0.15)	-0.30 (-0.61, 0.01)
**The DASH diet score**						
Overall score	**0.18 (0.01, 0.34)**	**0.85 (0.31, 1.38)**	0.15(-0.20, 0.50)	-0.01 (-0.15, 0.12)	**0.50 (0.10, 0.91)**	0.19 (-0.10, 0.49)
Language use	**0.17 (0.01, 0.33)**	**0.64 (0.13, 1.15)**	0.16 (-0.18, 0.50)	0.02 (-0.11, 0.15)	0.32 (-0.10, 0.74)	0.14 (-0.15, 0.44)
Media use	0.08 (-0.07, 0.24)	**0.89 (0.33, 1.45)**	0.07 (-0.31, 0.44)	-0.12 (-0.25, 0.01)	**0.58 (0.19, 0.98)**	0.25 (-0.05, 0.56)
Ethnic social relations	**0.29 (0.11, 0.47)**	0.30 (-0.09, 0.70)	0.09 (-0.19, 0.37)	**0.25 (0.09, 0.41)**	0.24 (-0.05, 0.53)	0.07 (-0.17, 0.32)
**Sedentary time**						
Overall score	0.05 (-0.03, 0.13)	**-0.72 (-1.00, -0.44)**	0.06 (-0.09, 0.22)	0.01 (-0.05, 0.07)	-0.19 (-0.40, 0.03)	-0.02 (-0.16, 0.12)
Language use	0.03 (-0.05, 0.11)	**-0.77 (-1.04, -0.51)**	0.10 (-0.04, 0.25)	0.02 (-0.05, 0.08)	**-0.30 (-0.52, -0.08)**	-0.01 (-0.15, 0.14)
Media use	**0.10 (0.02, 0.17)**	**-0.30 (-0.60, 0.001)**	0.02 (-0.15, 0.18)	0.03 (-0.03, 0.09)	0.01 (-0.20, 0.21)	0.02 (-0.12, 0.17)
Ethnic social relations	**-0.12 (-0.21, -0.04)**	**-0.34 (-0.55, -0.14)**	-0.04 (-0.16, 0.08)	**-0.14 (-0.21, -0.06)**	-0.06 (-0.21, 0.09)	-0.11 (-0.23, 0.01)
**Leisure-time physical activity**						
Overall score	**1.17 (0.31, 2.02)**	**7.54 (3.37, 11.72)**	**3.74 (1.61, 5.87)**	0.38 (-0.23, 0.99)	**6.62 (4.50, 8.74)**	**2.72 (1.10, 4.35)**
Language use	**0.86 (0.01, 1.71)**	**7.07 (3.09, 11.05)**	**3.24 (1.17, 5.31)**	0.24 (-0.36 0.85)	**6.35 (4.18, 8.53)**	**2.77 (1.15, 4.38)**
Media use	**0.82 (0.004, 1.63)**	3.53 (-0.85, 7.91)	**3.21 (0.93, 5.48)**	0.04 (-0.55, 0.62)	**4.31 (2.21, 6.40)**	1.46 (-0.22, 3.15)
Ethnic social relations	**2.16 (1.21, 3.10)**	**5.34 (2.31, 8.37)**	**2.11 (0.41, 3.81)**	**2.02 (1.30, 2.73)**	**3.93 (2.39, 5.48)**	**2.39 (1.03, 3.75)**

[a]Adjusted for age, marital status, income level, education level, and generation

Acculturation scores were converted into standard z-scores and beta coefficients were calculated for a 1-SD increase in acculturation scores
